# Relationship between menstruation status and work conditions in Japan

**DOI:** 10.1186/s13030-017-0112-x

**Published:** 2017-10-04

**Authors:** Mariko Nishikitani, Mutsuhiro Nakao, Shinobu Tsurugano, Mariko Inoure, Eiji Yano

**Affiliations:** 1 Institute of Decision Science for a Sustainable Society, Kyushu University, 3-1-1 Maidashi, Higashi-ku, Fukuoka-City, 812-8582 Japan; 2 Department of Psychosomatic Medicine, Teikyo University Hospital, Tokyo, Japan; 3 Graduate School of Public Health, Teikyo University, Tokyo, Japan; 4 Health Center, The University of Electro-Communications, Tokyo, Japan

**Keywords:** Female workers, Interval time, Long time work, Menstrual cycle, Anxiety for health

## Abstract

**Background:**

Menstrual problems can significantly impact daily and work life. In reaction to a shrinking population, the Japanese government is encouraging more women to participate in the labor force. Actual success in achieving this aim, however, is limited. Specifically, participation in the workforce by women during their reproductive years is impacted by their health, which involves not only work conditions, but also traditional family circumstances. Therefore, it is important to further assess and gather more information about the health status of women who work during their reproductive years in Japan. Specifically, women’s health can be represented by menstruation status, which is a pivotal indicator. In this study, we assessed the association between short rest periods in work intervals and menstruation and other health status indicators among female workers in Japan.

**Methods:**

Study participants were recruited from the alumnae of a university, which provided a uniform educational level. All 9864 female alumnae were asked to join the survey and 1630 (17%) accepted. The final sample of study participants (*n* = 505) were aged 23–43 years, had maintained the same job status for at least 1 year, and were not shift workers, had no maternal status, and did not lack any related information. The participants were divided into two groups according to interval time, with 11 h between end of work and resumption of daily work as a benchmark. This interval time was based on EU regulations and the goal set by the government of Japan. Health outcomes included: menstrual cycle, dysmenorrhoea symptoms, anxiety regarding health, and satisfaction in terms of health. Multiple logistic regression analyses were conducted to estimate the odds ratios (ORs) and 95% confidence intervals (CIs) for health indexes in association with interval time by adjusting for confounding variables that included both psychosocial and biological factors.

**Results:**

We compared the health status of women in the workforce with and without a sufficient interval time of 11 h/day. Workers who had a short interval time had a significantly higher prevalence of anxiety about health and dissatisfaction with their health. For menstruation status, only abnormal menstruation cycles were observed more often among workers in the short interval group than those of the long interval group. However, this association disappeared when biological confounding factors were adjusted in a multivariable regression model. Dysmenorrhea symptoms did not show a statistically significant association with short interval time.

**Conclusions:**

This study found a significant association between a short interval time of less than 11 h/day and subjective health indicators and the menstrual health status of women in the workforce. Menstrual health was more affected by biological factors than social psychological factors. A long work time and short interval time could increase worker anxiety and dissatisfaction and may deteriorate the menstrual cycle.

## Background

As a female-specific health indicator, a normal menstruation pattern is regarded as a pivotal indicator of women’s whole health status. Menstruation status is affected by biological factors [1, 2] and also psychological status [3]. Long hours at work are a source of occupational stress that increases psychological distress [4, 5], together with an increased risk of work-related injuries [6] and poor health outcomes [7]. However, hormones controlling the normal system of menstruation may alleviate the risk of cardiovascular disease for those in their reproductive years [8]. Therefore, menstrual problems can be considered as an important health indicator for female workers. A disordered secretion of these hormones due to work may impact women’s health and also increase risk to several chronic diseases, such as cardiovascular disease and metabolic disorder [8]. Thus, it would be meaningful to assess menstruation status among female workers.

Japan leads the world in dealing with an aging society and a declining birthrate. Thus, the Japanese government wants to encourage more women to participate in the labor force. However, our previous study demonstrated that there was no healthy workers’ effect among female workers in Japan [9]. In that study, we assessed lifestyles, knowledge and behaviors of healthcare, and subjective health status, but did not assess women specific health, like menstrual health status. In addition to the situation where many women resign upon giving birth, there may be other factors that influence the participation of women in the workforce in Japan. Looking at various specific health issues that impact women, such as menstrual cycle, may help to meet the goal of including more women in the workforce of Japan.

In Japan, despite an advanced educational background (43% in the prime age bracket have more than an upper-secondary education) [10], many Japanese women do not enter into economic activity. Although women’s participation in the workforce has increased recently, due in part to the Equal Employment Opportunity Law, revised in June 1985, more than half of working women are in precarious work positions that form the base of the gig economy, with part-time, short-term, unguaranteed, or outsourced work arrangements [11]. A precarious work arrangement reflects a lower work status for women because the economic rewards are inferior in terms of income and social security compared to that of regular workers [12]. In addition to their socioeconomic vulnerability, women’s status is unstable, with a higher frequency of leaving jobs, and changing workplaces. Therefore, it is difficult to determine the health of female workers, except for those in certain particular occupations, such as nurses. Thus, it is important to pursue further research regarding work conditions and health for women in the workforce.

Worker health, when considering gender differences, is affected not only by work conditions, but also family circumstances. An imbalance between work and family demands may be a strong risk factor for female workers [13], and it has been suggested that the younger generation especially are tasked with the multiple roles of housework and mother [14] than male are workers. Meanwhile, some argue that male workers are subjected to more work stress that induces lifestyle diseases and mental disorders.

The hypothesis of our study was that female workers spending a longer time at work have more problems with menstruation and other health status items than do female workers working shorter times. Along with other developed countries, the EU (European Union) has indicated an interval time to regulate work conditions, with a minimum daily rest period of 11 consecutive hours over every 24 h, to control excessive work and guarantee sufficient time for rest [15]. In Japan, long work hours are debated as a social issue [16], and consequently, the MHLW has introduced this regulation as a challenge to employers to apply workplace policies that provide for a continuous rest time of 11 h or more over a 24-h period, and this is called Interval Time (“*Kinmu Kan* Interval”) [17]. In this study, we assessed whether such an interval time was effectively in place for women at work and possible effects on their health status.

## Methods

### Study participants

Study participants were recruited from the alumnae of a national university located in Tokyo to produce a sample with a uniform educational level and a relatively common family background. From February to May 2007, we distributed a notice regarding the survey purpose and a policy about privacy protection to all 9864 female alumnae. We requested informed consent and received agreement/consent cards from 1630 (16.5% response rate), and then we mailed self-administered questionnaires. We received 1515 responses (valid response rate, 15.4%). Of these respondents, 411 alumnae did not engage in paid work, and were students, housewives, unemployed, etc. Among those remaining, 53 alumnae had started work within the last year, 143 alumnae engaged in shift work, 135 alumnae did not provide enough information about work and commuting hours, and 126 alumnae were over 43 years old or lacking age information (*n* = 3). The age 43 was decided upon as a cut-off based on previous studies about average menopausal age [1, 2]. Moreover, among the remaining alumnae, 80 who were pregnant, in a period of lactation, in menopause, or taking contraceptive pills at the survey point and 62 who did not provide a complete set of information for the variables used in this study analysis were excluded. The final number of study participants was 505. Prior to survey distribution, the Institutional Review Board of Teikyo University School of Medicine approved the study.

### Work conditions: Interval time

To assess the association between hours worked and health status, we divided the participants into two groups according to interval time. The interval time in this study, hours between being off work and resuming daily work, was calculated by participant information described in the questionnaire about average regular work time (hours/month) and average commute time (hours/day). We assumed that participants engaged in work 20 days per month, added commute time and daily work time including extra work time, and then calculated the average interval time (hours/days) by subtracting these total hours from 24 h. Based on the basic idea of the Work Time Directive by the EU [15], we set 11 h or more as a sufficient interval for hours off work, and less than 11 h per day as insufficient time off work.

### Health indicators: Menstruation and subjective status

The study participants were expected to be generally healthy and their cooperation with this study was solicited via mail, thus we used subjective health indicators in the questionnaire. We asked participants to answer about the regularity of their menstrual cycle and the recent average period of one cycle. An abnormal menstrual cycle was defined as either an answer of irregular cycle or an answer about one cycle as being less than 24 days or more than 39 days for each regular cycle based on the definition by the Japan Society of Obstetrics and Gynecology [2]. For dysmenorrhoea symptoms that occurred before or during menstruations, we defined this as answers indicating a level of decrease in work efficiency due to dysmenorrhoea symptoms among those who answered that they experienced unpleasant symptoms due to menstruation. Because study participants were asked, “Do you experience unpleasant symptoms, like abdominal pain, back pain, head ache, any painful symptoms, breast tenderness, irritability, food craving, episode of diarrhea, nausea, and drowsiness etc.?”, we defined this indicator as also including premenstrual symptoms. Study participants were also asked about taking pain relief medicine and seeing a doctor for these unpleasant symptoms. Anxiety regarding health (general, physical, mental, and any others) was addressed to screen for symptoms of mental problems, which can be common among those of working age, such as depression [16, 17]. Based on previous studies [18–20], satisfaction in terms of health (well-satisfied and satisfied vs. not very satisfied and unsatisfied) was explored because level of health satisfaction was expected to predict comprehensive health associated with lifestyle and sociodemographic characteristics [21].

### Other information: Lifestyle, family, and job status

Respondents were asked to report age, height and weight, present smoking habit, what they ate for breakfast and how frequently they ate breakfast each week. The diet information was summarised as a binary variable: consumption of a staple food and main dish every morning (sufficient breakfast) vs. no such consumption. In addition to the above-stated items, family demands (marital status and family members) [9], educational background (graduate school or under graduate), the type of job (regular employment or not), subjective socioeconomic status (upper, upper-middle, middle, lower-middle, or lower), the length of time at their present job status, and their occupation were asked. Moreover, items for exclusion criteria, such as pregnant, lactation, menopause, taking contraceptive pill, and shift-work status, were confirmed on the questionnaire.

### Statistical analysis

The first step in the statistical analysis was to obtain frequency counts (categorical variables) or median and 25th–75th percentiles (the distributions of continuous variables were not assumed to be normal) of all variables of interest for the three groups according to interval time. Second, a *chi*-square test was used to compare the housewife group with the other two groups in terms of categorical variables, and a Wilcoxon rank-sum test was used to compare the groups with respect to continuous variables.

Finally, multiple logistic regression analyses were conducted to estimate the odds ratios (ORs) and 95% confidence intervals (CIs) for health indexes in association with interval time. We calculated crude ORs, and then estimated ORs by adjusting for confounding variables of psychosocial factors, including environmental ones such as social support, biological factors, and both types of factors. As important confounding variables of psychosocial factors, family care demands (binary, living with child/children, husband, and elderly parents, or not) [9], and satisfaction with present social status (binary, Yes or No) were used. As additional important confounding variables, biological factors, age (five were categorised variables according to percentile values: 20%, 28 years old; 40%, 32 years old; 60%, 36 years old; and 80%, 40 years old), smoking habit (binary, Yes or No), body mass index (BMI; five categorised variables according to percentile values: 20%, 18.4 kg/cm^2^; 40%, 19.4 kg/cm^2^; 60%, 20.3 kg/cm^2^; and 80%, 21.7 kg/cm^2^), dietary habit (binary, having a well balanced breakfast every morning, or not), and parity experience (binary, Yes or No) were used.

Data analyses were conducted using STATA (version 13, State Co, College Station, TX, USA). All tests were two-sided, and a *p*-value <0.05 was considered significant for the ORs.

## Results

### Basic characteristics of study participants

The basic characteristics of the 505 study participants are shown in Table 1. Workers who have less than 11 h of interval time between finishing and starting work, including commute time, (short interval group) were only 37 (7.3%), and workers who have 11 h and more time as an interval (long interval group) were 468 (92.7%). Most workers of the short interval group were younger (median age, 29 years old) and more were unmarried (*n* = 28, 76%) than were those of the long interval group (media age was 33 years old, and 219 (47%) were unmarried). Most of the participants have never experienced delivery and currently live without children (both *n* = 32, 86%). Although their length of time in the present work status was less than that of workers of the long time interval group (median length 3.9 years, vs. 6.7 years), most were engaged in their job as regular employees (n = 32, 86%). Almost half (48%) were engaged in professional and highly technical jobs and were teachers at various types of school, such as elementary, junior high, and high school, university, and graduate school, but there was no significant difference to that of the long interval group. From these situations, aside from patterns reflecting the occupations, the participants in the short interval group might have little social support, such as through partners, and may have long work times because of regular employment. Thus, their work-related times, such as regular work time, extra work time after regular work, and commuting time, were all longer (14.5 h/day) than in the long interval group (9.7 h/day).

**Table 1:** Basic characteristics of 505 study participants by interval time

Number (%) or median (25–75% range)			Less than 11 h of interval time (*n* = 37)	11 h and more interval time (*n* = 468)	*p-*values^a^
Age (years)			29 (25–38)	33 (29–38)	*0.0101*
Smoking (yes)			1 (3%)	19 (4%)	*0.684*
Body mass index (kg/m^2^)			19.9 (19.4–22.3)	19.8 (18.6–21.2)	*0.1614*
Insufficient breakfast (not a well-balanced breakfast or not every morning)			18 (49%)	264 (56%)	*0.360*
Parity (yes)			5 (14%)	155 (33%)	*0.014*
Family care demands (yes)			12 (32%)	265 (57%)	*0.004*
	Living situation (alone)		10 (27%)	116 (25%)	*0.762*
	Marital status (married)		9 (24%)	249 (53%)	*0.001*
	Living with children (yes)		5 (14%)	161 (34%)	*0.009*
Satisfaction with present social status (Satisfied)			31 (84%)	383 (82%)	*0.767*
	Education (graduate school)		12 (32%)	168 (36%)	*0.672*
	Regular employment (yes)		32 (86%)	315 (67%)	*0.015*
	Subjective economic conditions (Upper & Upper middle)		17 (46%)	177 (38%)	*0.328*
	Length of time in present status (years)		3.9 (2.7–10.7)	6.7 (3.2–11.8)	*0.1634*
	Occupations:	Clerical and related workers	13 (35%)	177 (38%)	*0.213*
		Professional and highly technical job	6 (16%)	133 (28%)	
		Teachers at school	12 (32%)	95 (20%)	
		Others	6 (16%)	63 (13%)	
Average interval time (hours/day)			9.5 (8.4–10.3)	14.3 (12.8–16.4)	*<0.0001*
	Average regular work time (hours/month)		250 (240–280)	170 (140–200)	*<0.0001*
	Average extra work time (hours/month)		50 (40–90)	11 (0–34)	*<0.0001*
	Average commuting time (hours/day)		1 (1–2)	1 (0–2)	*0.0023*

^a^Comparison by chi-square test, and Wilcoxon’s rank sum test

### Menstruation health and subjective health status

The prevalence of an abnormal menstrual cycle was significantly higher in workers of the short interval group (*n* = 13, 35%) compared to the long interval group (*n* = 95, 20%) (Table 2). Workers who answered affirmatively to the decrease in work efficiency for dysmenorrhea symptoms did not show a significant difference between both groups. There was no significant difference in the individual anxiety point, and more workers of the short interval group answered yes for anxiety about any health status (*n* = 22, 60%) than did those of the long interval group (*n* = 181, 39%). In addition, the prevalence of health dissatisfaction was significantly greater among workers of the short interval group (*n* = 15, 41%) than among those of the long interval group (*n* = 109, 23%).

**Table 2:** Health status of 505 study participants by interval time

Number (%)	Less than 11 h of interval time (n = 37)	11 h and more interval time (n = 468)	*p-*values^a^
Abnormal menstrual cycle (24 >, >39 days / one cycle)	13 (35%)	95 (20%)	*0.034*
Decrease in work efficiency due to dysmenorrhoea symptoms	22 (60%)	240 (51%)	*0.338*
(Among the above subjects) pain relief medicine for these symptoms	12 (55%)	136 (57%)	*0.848*
See doctor for these symptoms	2 (9%)	64 (27%)	*0.065*
Anxiety about health	22 (60%)	181 (39%)	*0.013*
(Among the above subjects) about physical points	15 (68%)	117 (65%)	*0.742*
About mental points	5 (23%)	60 (33%)	*0.322*
About other things	2 (9%)	3 (2%)	*0.092*
Dissatisfaction with health	15 (41%)	109 (23%)	*0.019*

^a^Comparison by chi-square test, and Fisher’s exact test

Table 3 shows the risks for poor health status in workers of the short interval group as crude ORs and adjusted ORs for confounding variables of biological and psychosocial factors. Abnormal menstruation cycles, anxiety about health, and dissatisfaction about health had a significantly higher odds ratio among workers of the short interval group. Abnormal menstruation cycles remained at a higher odds ratio when adjusted for psychosocial confounding factors (OR = 1.90, *p* = 0.085), but a greater decreasing tendency of OR was shown when biological confounding factors, such as age, smoking, BMI, dietary habit, and delivery experience were introduced in the regression models (biological factors adjusted model, and all adjusted model). The risk of decrease in work efficiency for dysmenorrhea symptoms did not show a significant OR, although it indicated an increased tendency of health risk. Two subjective health statuses, anxiety about health and dissatisfaction with health, remained significant after being adjusted for any confounding variables.

**Table 3:** Health risk of short interval time (less than 11-h per day) for working women (n = 505) in Japan [odds ratios (ORs), 95% confidence intervals (CIs), and *p*-values]

Crude odds ratios^a^, 95% CIs	Crude ORs (95% CIs) *p*-values	Adjusted ORs (95% CIs) *p*-values		
		Psychosocial factors adjusted model^a^	Biological factors adjusted model^b^	All adjusted model^c^
Abnormal menstrual cycle (24 >, >39 days / one cycle)	2.13 (1.04–4.33) *0.038*	1.90 (0.92–3.95) *0.085*	1.77 (0.85–3.71) *0.128*	1.77 (0.84–3.74) *0.134*
Decrease in work efficiency for dysmenorrhea symptoms	1.39 (0.71–2.75) *0.340*	1.32 (0.66–2.63) *0.428*	1.18 (0.59–2.39) *0.640*	1.21 (0.60–2.45) *0.599*
Anxiety about health	2.33 (1.18–4.60) *0.015*	2.20 (1.10–4.40) *0.026*	2.08 (1.04–4.16) *0.039*	2.07 (1.03–4.17) *0.042*
Dissatisfaction with health	2.25 (1.13–4.48) *0.022*	2.19 (1.08–4.45)* 0.029*	2.05 (1.01–4.13) *0.046*	2.05 (1.01–4.18)* 0.048*

^a^Adjusted for family care demand, and satisfaction with present social status

^b^Adjusted for age, smoking, BMI, dietary habit, and parity

^c^Adjusted for age, smoking, BMI, dietary habit, parity, family care demand, and satisfaction with present social status

## Discussion

In this study, we compared the health status among female workers with and without a sufficient work interval time between being off work and resuming their daily work, at 11 h/day. This metric is based on a regulation by the EU and the goal set by the government of Japan. Workers who had a short interval time of less than 11 h/day showed a significantly higher prevalence of anxiety about health and dissatisfaction with their health. For menstruation status, only abnormal menstruation cycles were observed as more prevalent among workers in the short interval group compared to the long interval group. However, this association disappeared when biological confounding factors were adjusted in the multivariable regression model. Dysmenorrhea symptoms that decrease work efficiency did not show a significant association with less than 11 h of interval time.

Abnormal menstrual cycles, a particular health status factor for women, can be impacted by an insufficient amount of rest, and the prevalence showed a tendency toward a positive association with a short interval time. However, the association was weaker than those of subjective and psychological health indicators such as anxiety and dissatisfaction with health. Moreover, when biological factors were adjusted in the risk assessment model, menstrual cycle was not an influential factor. Menstrual cycle is a kind of biological clock [22] that is mainly regulated by hormonal secretion in cooperation with the hypothalamus-pituitary-ovary axis. Therefore, it is likely that deterioration of the menstrual cycle according to a short interval time has a complex mechanism.

The interval time between being off work and resuming daily work is considered to be a substitute for total labor hours. At the same time, it also can include late-night and/or early-bird shifts at work. We excluded shift time workers (*n* = 143, about 14% of workers who have kept their job in the same style for the last 1 year) from analysis in this study, but possible long labor hours among the short interval group in this study might be similar to shift work. In fact, several studies reported that shift work impacted circadian rhythms [23, 24], and a study assessing Chinese female nurses observed the effect of rotating-shift work on irregularity in menstrual cycles [25]. A systematic review affirmed the effect of shiftwork on menstrual disruption, not only in terms of abnormal cycles, but also spontaneous pregnancy loss [26]. Another study showed that night work itself did not show a significant association with an irregular menstrual cycle [27]. Therefore, future research with a detailed study design is warranted to assess the effect of interval time on female health.

Dysmenorrhea symptoms, another health status factor for women, did not have a steady association with interval time. The reason for the lack of a significant association with menstruation indicators might be the same as for menstrual cycles that are affected strongly by biological factors. The lack of statistical power was true for these weak associations, because the number of workers of the short interval time group was very small (7.3% of total study participants, *n* = 505). More than anything else, menstrual cycle disorder is more easily recognizable than dysmenorrheal symptoms, because the former is the result of a day count, and the latter is often associated with pain. If workers recognize an impact to their health, it may be possible to change the work interval time.

Anxiety about health was an important subjective symptom among the working-age population because this cohort was exposed to many sources of psychological distress [18]. In addition, anxiety and depression are common diseases among women of a reproductive age [28–32]. These psychological disorders can occur with pregnant and postpartum women, and one possible mechanism may come from imbalances in hormone excretion. Previous studies about shift-work workers suggest a negative effect of work during the night that confuses the circadian rhythm and causes mental health issues such as insomnia, anxiety, and depression [23, 24]. The short work interval time in this study could also cause such mental health problems among workers.

We used dissatisfaction with health in this study to explore the comprehensive life status of the participants. According to a previous study, satisfaction with health was predicted by symptoms and present medication [19]. A recent study found an association with life satisfaction [20]. Both studies observed a steady association between satisfaction and health and health-related behaviors, called good practices, such as having exercise, no snacks, and maintaining a good BMI. Therefore, the positive association of health dissatisfaction with a short interval time of less than 11 h/day indicated in this study may reflect a deterioration of worker quality of life. Together with anxiety, a short interval time could have a deleterious effect on the psychosocial health status of workers.

This study has several limitations. First, all variables were measured thru self-reports. According to other questionnaire-based research that relied on mailed surveys or interviews, the observed result should be interpreted as in the range of subjective health status. Thus, classification errors were likely because we divided the participants into short and long interval time groups based on self-reported answers; They were not derived from actual data regarding attendance at each workplace.

Second, all participants were adult women who had graduated from a university, which may have affected the generalisability of the results because such people often come from higher-income families [33] and could engage in occupations with better conditions, such as those that are more discretionary, controllable, less demanding, and of a higher income than other typical occupations. In fact, the labor participation rate was 78.6% in this study population, which was much higher than that of women overall in Japan, which is 48.4% in statistical estimates by the government [11]. Importantly, the comparisons here and in following comparisons should be done carefully because the age distribution was different. The study participants were rather younger than those in the governmental statistics. In addition, the percent of regular employment of this study population was higher (72.0%) than that from governmental estimates (44.7%). Most of the study population (73.5%) were engaged in specialist occupations and highly technical jobs, including as teachers in elementary school to graduate school. Thus, it was difficult to say that they were representative of the typical job status of Japanese women overall, where the percent of these occupations has been estimated at 16.4%. Thus, the observation in this study from the view point of health status and the effect of short interval time might be further weakened by such advantageous working conditions. Overall, however, our results can be generalised as representative of well-educated women, a group whose numbers are expected to increase in the future.

We recruited study participants by asking for voluntary participation, and 16.5% of the candidate subjects showed a willingness to participate. Moreover, the final response rate was 15.4%. Thus, from the perspective of selection bias, the study participants may have held positive attitudes towards work and health and be more health conscious than those who did not participate, because limited information about the study purpose was given and several key words were provided, such as employment status and health, in our recruitment efforts. The effect of these attitudes is ambiguous, and our findings might be biased by it. Such self-selection bias could work in both directions, and it would not be evident which effect was stronger.

Moreover, we did not use standardized psychological scales to assess the relationship between interval time and a worker’s metal health status. This analysis was a second attempt to use the same dataset [9], and the first objective of this survey data had focused on employment status and general health status, including lifestyle and health knowledge, and was not focused on mental health, and thus employed different scales. In future studies, it would be better to use standardized psychological scales for assessment of the health effect of interval time.

Finally, our study design was neither longitudinal nor interventional. A cross-sectional study cannot identify a causal effect relationship between work conditions and health status. Therefore, further studies are needed to better validate the effect of interval time on workers’ health.

## Conclusions

This study found a significant association between a short work interval time of less than 11 h/day and subjective health indicators and the menstrual health status of women. Menstrual health was more affected by biological factors than by social psychosocial factors. Long work time and a short interval time could increase worker anxiety and dissatisfaction, which might impact the menstrual cycle.

## Abbreviations

BMI: Body mass index
CI: Confidence interval
EU: European Union
OR: Odds ratio
